# The Composer’s Program Note for Newly Written Classical Music: Content and Intentions

**DOI:** 10.3389/fpsyg.2016.01707

**Published:** 2016-11-09

**Authors:** Diana M. Blom, Dawn Bennett, Ian Stevenson

**Affiliations:** ^1^Music, School of Humanities and Communication Arts, Western Sydney University, SydneyNSW, Australia; ^2^Research and Graduate Studies, Curtin University, PerthWA, Australia

**Keywords:** new music, program note, performer, listener, practice-based research, artistic research, audience

## Abstract

In concerts of western classical music the provision of a program note is a widespread practice dating back to the 18th century and still commonly in use. Program notes tend to inform listeners and performers about historical context, composer biographical details, and compositional thinking. However, the scant program note research conducted to date reveals that program notes may not foster understanding or enhance listener enjoyment as previously assumed. In the case of canonic works, performers and listeners may already be familiar with much of the program note information. This is not so in the case of newly composed works, which formed the basis of the exploratory study reported here. This article reports the views of 17 living contemporary composers on their writing of program notes for their own works. In particular, the study sought to understand the intended recipient, role and the content of composer-written program notes. Participating composers identified three main roles for their program notes: to shape a performer’s interpretation of the work; to guide, engage or direct the listener and/or performer; and as collaborative mode of communication between the composer, performer, and listener. For some composers, this collaboration was intended to result in “performative listening” in which listeners were actively engaged in bringing each composition to life. This was also described as a form of empathy that results in the co-construction of the musical experience. Overall, composers avoided giving too much personal information and they provided performers with more structural information. However, composers did not agree on whether the same information should be provided to both performers and listeners. Composers’ responses problematize the view of a program note as a simple statement from writer to recipient, indicating instead a more complex set of relations at play between composer, performer, listener, and the work itself. These relations are illustrated in a model. There are implications for program note writers and readers, and for educators. Future research might seek to enhance understanding of program notes, including whether the written program note is the most effective format for communications about music.

## Introduction

The written program note for music heard in concerts of Western classical music has served as a guide to listeners since at least the 18th century (Scaife, 2001). The program note takes the form of a short text about a musical work, often containing historical context, composer biographical details and compositional thinking. It can, in reality, be viewed as a form of analysis of the work. While primarily intended for the listening audience, the program note can also be placed in a score along with technical performance notes for the information of the performer. Despite the program note’s long history, little research has investigated this way of writing about music. The assumption is that the program note provides a benefit to listeners and performers in that it may enhance their understanding and experience of the musical work.

Three factors guided the focus of this exploratory study into composers’ intentions when writing a program note about their own music. The first of these relates to the authorship of program notes; while program notes are often written by professional program note writers, performers and concert organizers, composers of contemporary classical music tend to be asked to write a note about their own works. The second guiding factor concerns the extant literature, which has focused on program notes in relation to canonic works and has, to this point, ignored contemporary classical music. Finally, the three researchers are actively engaged in contemporary classical music as practitioners—composers, performers, and artist educators—and as such we were uniquely positioned to undertake the study from the multiple perspectives of practitioner and researcher (Nelson, 2013).

In particular, the study sought to understand, in a more systematic way, the intended recipient, role and the content of composer-written program notes. Seventeen contemporary composers, all of whom had written musical works for one of our three contemporary classical music projects, were asked to respond to a series of questions about their thinking on program note writing. Recognizing several gaps in the literature, we sought information on the composer as program note writer about their own newly composed works; the role the composer would like the program note to play; and the type of information required to achieve this role. Three research questions were posed:

(1) For whom does a contemporary classical composer write a program note?
(2) What is the intended role of the program note?
(3) What information does the composer want to communicate about their newly composed works?

## Literature

Because of the lack of extant research into program notes *per se*, we sourced a wide range of writings around the topic. This included studies on composer intentions, program notes for the music of composers who are no longer living, and listeners or audiences and the concert experience. We also consulted educational writing for higher education performers on how to write program notes. Finally, we considered articles on the thinking of composers and other people involved with new music festivals. These articles included written reflections from one professional program note writer, and in one instance we drew on a team member’s personal experience as a composer.

### Empirical Research into Program Note Intention

Our study responded to Ferrara’s (1984) call to unlock the composer’s intention by seeking an understanding of what information might be given to the listener/performer as a written note and what might be left to the language of music itself. Drawing on phenomenology as a tool for musical analysis, Ferrara observed that “at both the composing and interpreting stages, music is imbued with a human presence … marked by the historical *being there* of the composer” (p. 357). On this basis, while it may not be possible to know a composer’s intention, “it is necessary to understand a work within the perspective of the world in which it was written” (p. 357).

In Ferrara’s (1984) analytical framework, developed to give a phenomenological analysis of *Poème électronique* by Edgard Varèse, Ferrara considered what listeners hear when exposed to multiple renditions of a work. He concluded that listeners first observe only the *syntactical* meanings of the work: they consider sounds in relation to their individual and connecting phonemic qualities. At the next level, listeners make *semantic* meanings by interpreting sounds and making sound relationships, from possible references to complex understandings of the sounds within a work. The most complex listening involves listeners who are able to make *ontological* meaning of the music through socially constructed relationships between the music and their own life world contexts.

The ontological meaning, then, takes into account the work in its entirety, which “in some works presents a glimpse of the historically based [onto-historical] world of the composer” (Ferrara, 1984, p. 357). The relevance to our program note research is that at the time of composing a work there is a “lived time” specific to that composition style and other contextual elements, and yet for much music the listener, performer or analyst must create their own meaning in another time and/or place. Of interest here is the extent to which composers in our study intended, through their program notes, to interject their own time and context into the experience of the listener such that “onto-historical existence is grounded in the work and may be ‘preserved’ by the listener of the future” (Ferrara, 1984, p. 372). After his analysis of the Varèse work, Ferrara argued that analysis and musical work are not two separate entities; rather, “each emits and resonates meanings that intersect in an ideational space” (p. 373). If the program note is similarly positioned as a form of analysis, then Ferrara’s comment is also applicable to this form of writing.

In the only previous study on the program note, Margulis (2010) employed excerpts from Beethoven string quartets to understand listeners’ reactions to written program notes. Beethoven’s music, although contemporary in its frequent appearance in concert programs, is not recently composed; however, the discussion and focus on program notes is of interest to our study. In particular, we were keen to make use of Margulis’s definition of two types of program note text:

(1) Dramatic notes are those that use emotive, often pictorial language to describe a scene or sensation: for example, a storm at sea with rolling waves, violent winds and crashes of thunder; and
(2) Structural notes describe compositional aspects of the work, which may include the form, structure, or striking timbral qualities.

Contrary to Margulis’s hypothesis, listeners’ enjoyment of the music was not heightened by text descriptions provided in advance of their listening. Moreover, she found that listeners’ enjoyment was less when they received dramatic program notes than when they received notes that were structural. Although Margulis’s research concerned listeners rather than performers and composers, the positive and negative impact of programs notes has been echoed in research that examines the experiences of performers and their relationship with program notes. For example, in their search to understand a stylistically unusual and conceptually challenging work by Australian composer Ross Edwards, pianists Viney and Blom (2015) noted a lack of non-musical (ontological) or programmatic information in the score of *Kumari*. For Blom, it was structural information that provided the key to understanding the work and allowed a deeper rehearsal process to be undertaken; for Viney, it was ontological information.

In another performance-based study, Bennett and Blom (2014) recorded the process of building their collaborative interpretation of a newly composed work. The duo found that when composer-pianist Blom gave dramatic program note information to violist Bennett halfway through the rehearsal period, there was a misinterpretation of the work because of what Margulis called “unique images” (p. 295) in the form of dramatic descriptions. In this case, the unique images stemmed from the link to a previous composition, and despite further discussion, rehearsal, performances and the recording of the work, neither performer has entirely forgotten the viola player’s initial response. Here, the composer felt she had given too much information in the program note, with the result that the violist was unable to move past the literal description.

### Writings by the Users and Writers of Program Notes

The program note in written form arose from the migration in music performances from the private salon to the public concert hall, at which time listeners expressed an interest in “printed explanations and instructions” (Bebbington, 2004, p. 1). Views on the intent and content of the program note appear in a range of writing beyond the scholarly literature. Professional program note writer Burkat (1985, p. 1), for example, warned of the dangers of incorrect or overly technical information being included in a program note because it can “become fact, passed on for example from written document to radio program and back to document.” When writing a note for a newly composed work, Burkat’s strategy was to contact the composer and ask what he or she would like to be written about the piece. Burkat (1985) found that “sometimes the composer can express thoughts and feelings in words so precisely and effectively that no one else’s [words] will do” (p. 2). However, he also noted that some composers had nothing to say about their work “except technicalities that will mystify the lay listener and distract attention from the music itself” (p. 2).

The technicalities to which Burkat refers are consistent with Margulis’s “structural” comments. At odds with Margulis, Burkat advocated that listener enjoyment would be rated more highly when dramatic, rather than structural notes, were provided. This may of course differ according to the particular demands of hearing atonal and/or complex new music, but it might also expose the belief, noted earlier, that a dramatic program note enhances the listening experience. A negative view of structural information appeared in a letter to the editor of a composers’ association newsletter in which Hinds (2014, n. p.) noted concern with the lack of listener appeal in some contemporary classical compositions and bemoaned that many program notes accompanying such works “are replete with mathematical analysis, but little on the deeper meaning of the works.”

Composer Smalley (1997), arguing for his development of the concepts and terminology of *spectromorphology* as tools for describing and analyzing the electroacoustic listening experience, acknowledged that composer communications are not always beneficial to the listener:

What the composer has to say (in program notes, talks, sleeve notes) is not unimportant, and it undoubtedly influences (both helping and impeding) the listener’s appreciation of music and musical ideas, but it is not always perceptually informative or relevant. (p. 107)

Also writing of the electroacoustic listening experience, Landy (2007, pp. 21–62) argued that carefully prepared dramaturgical (dramatic) information regarding a composer’s intentions can provide listeners with “something to hold on to,” guiding the listener and helping to avoid the sometimes alienating experience of listening to the unfamiliar sound-world of electroacoustic music. Landy (2007) proposed that such an approach is essential to engaging the listening audience and overcoming the marginalization of contemporary music. French composer Jean-Claude Risset, whose music embraces electronics and acoustic instruments, agreed with this sentiment. Risset (Cochrane, 2013, p. 29) suggested that a detailed narrative might enable listeners to “relate in some way to the music, even if it was not the way I intended”.

Festival attendee and writing mentor Matthew Lorenzon (2015) bemoaned the lack of a printed program at the 2015 Australian Bendigo International Festival of Exploratory Music, noting that several performers introduced musical works themselves; however, not every concert organizer feels that program notes are necessary or even desirable. Composer Diana Blom, one of the research team, had written of her experience as a composer at a festival of new contemporary classical music in Italy, where works were listed but no written program note information given. In this case, “festival organizers … were adamant that the listeners/audience should make up their own minds about the new works and not be guided at all by a program note“ (personal communication, 2016). This was a deliberate strategy to encourage listeners to hear the music without external influence; yet post-concert discussion with listeners and composers had revealed that many would have liked some form of program to know more about the works.

The final source of information about the nature and function of program notes is that of guides written for higher education students who are required to create program notes for their performances. Bebbington (2004, p. 1) created two such documents for students and noted the inappropriateness of “expansive scholarly study peppered with footnotes or an in-depth analysis.” Bebbington’s (2004) concern related in part to the difficulty of reading small text in the low lighting of many concert halls; however, he also focused on the intent of program notes, which he argued is “to concisely inform listeners about the music they are hearing and to assist them in its direct appreciation” (p. 1). As such, he asserted, the program note should take “no longer to read than the piece does to play … and ideally much less” (p. 1).

Concerned about dense or lengthy program notes that divide the attention of the listener and frustrate their enjoyment of a work, Bebbington (2004) encouraged students to use the tools and resources of the musicologist and to give listeners “at least two pieces of information that will help them understand what they are hearing, and two or three salient features to listen out for” (p. 4). He suggested explaining a descriptive title, the background of the work in relation to “how and when it came to be composed” (p. 4), and the context of the work in relation to “the historical idea, the artistic trend, or the literary or artistic or philosophical movement which produced it, or the cultural milieu from which it comes” (p. 4). While this is largely ontological information, Bebbington also encouraged salient semantic and/or syntactical features alongside some dramatic writing about the title, where relevant.

Pianist-educator Scaife (2001) noted the 200-year history of the “annotated program,” and he included under the label of program notes such writings as CD notes and notes found on the Internet. Scaife (2001, p. 3) recommended that Diploma performance candidates consider three groups of people when writing their program notes: first, the examiner, to express “how well you understand the musical and historical context of the repertoire”; second, the audience, to increase listeners’ appreciation and enjoyment; and third, the performer, to help “clarify thoughts about the music that they are to perform.” The marking rubric for program notes at Diploma distinction level looks for, among other things, notes that are “pertinent and persuasively written … with a well-balanced commentary” (p. 4). Much advice is given on structure and content, performance practice (interpretive) issues, and background reading.

Like Burkat (1985) and Scaife (2001) urged students to double-check their facts and draw their own conclusions as information can be unreliable. Scaife drew on the writings of music critic Turner (1933), who found both the “purely descriptive and the purely technical-analytical” program notes to be “objectionable and useless,” and that the overuse of dramatic writing with its metaphor and emotion is “useless to everybody, and positively harmful to those who are seriously trying to understand the art of music” (in Scaife, 2001, p. 7). For Scaife (2001, p. 8), the program note exists to help the listener or reader “develop an understanding of the music”; therefore, it is useful to include some biographical and historical information, and to explain how the work reflects the aesthetic tradition in which it exists (ontological). However, reminiscent of the experience of Bennett and Blom (2014) described earlier, Scaife (2001, p. 8) also warned against revealing too much of the “human story behind a piece of music.”

Scaife’s examples of structural and syntactical information include some semantic statements, and in his two examples of best practice (for works by Janáček and Duparc), Scaife provided a balance of ontological, dramatic, semantic, and structural information. Of interest, Scaife’s examples were written in the third person and did not include the personal voice of the performer; nor do the guides refer to writing notes for the works of living composers. However, there is a common call for accurate information, balance of content, a warning about too much structural information, and advice against including personal opinion.

There is a lack of extant research into program notes *per se.* Why this is so is beyond the scope of this study but the assumptions and traditions of program notes, mentioned earlier, have perhaps presumed that the listener, composer, performer, and concert organizer are all happy with the status quo. The diverse literature reviewed above indicates that is not always so. However, with only one exception the program note is positioned, for both canonic and contemporary classical music, as an informative and positive inclusion, with differing advice on content style and acknowledgment of the program note writer as the professional, the student, the performer, and the composer. Our study aimed to narrow the writer to the contemporary classical composer and investigate further the issue of content style and issues of what this communicates.

## Materials and Methods

The participants, 17 composers, had written new works for one of three new music projects. The projects were embedded in research-based practice and each resulted in one or more public performances and academic articles, and a commercial recording ^1^. *Antarctica* resulted in works for one or two instruments played by one pianist/performer; *Australia East and West* sought works for viola and piano; and *Playing with Fire* engaged composers with electroacoustics (CD soundbed, delay, beat sequence, electro-acoustics) and live acoustic piano. The first project included composers from Australia, New Zealand, Italy, Portugal, and Spain, and composers involved in the other projects lived in Australia. Twelve composers were male and five were female; they were all professional composers and ranged from early careerists to established careerists. All participating composers had written at least one program note for the works contributed to our projects; however, the study focused more broadly on the program notes composers had written for their works in general. Once ethical consent was obtained from the lead university, composer participants across the three projects were invited to complete a questionnaire on their program note writing; this was distributed by email once each composer had submitted their composition. Participants signed a consent form and they were assured of their anonymity. The first 12 composers responded to questions one and two about their written program notes, and initial analysis revealed that they were also disclosing facets of their program note process and content. To learn more about this, we posed two additional questions (questions 3 and 4). The final five composers answered all four questions.

Please answer the following questions in relation to your recent experience composing for the project (one of the three projects named above):

(1) What role should a program note play for the performer and the listener?
(2) Should different information be given to the performer and the listener?
(a) If different, what sort of information should be given to each?
(3) How do you go about writing a program note?
(4) What do you include, and why?

We employed a “naturalistic” coding process that started with reading each response without applying codes. Following Glaser and Strauss (1967), we then used a constant comparative analytical scheme that involved unitizing and categorizing the text, which was broken into units of information. These units were subsequently brought together into provisional categories relating to the same content. Next, deductive analysis was conducted using Ferrara and Margulis’s frameworks to classify information that might be given to the performer and listener and to categorize the responses. The final dataset was then organized into themes for discussion.

Throughout the analytical process, two primary coders were used to reduce error and bias in coding the responses (Mays and Pope, 2000). There were eight rounds of discussions between the coders, and the third coder coded all instances of disagreement prior to further discussion. The analysis moved gradually to higher levels of abstraction, moving from a close association with individual cases toward a concern with broad analytic themes.

Finally, data were displayed in a way that is conceptually pure, making distinctions that are meaningful and which provide interesting content. This included detailed discussion of multiple themes complete with subthemes, illustrations, quotations and multiple perspectives from different respondents.

We note that one performer (P1), a pianist, chose to respond to the composer questions when copied in to the questionnaire email. Her responses, while not included in the composer data, are included in the findings where relevant as they give a complementary performer’s perspective. The composers are identified here using a code based on gender, project (Antarctica: A; Australia East and West: EW; Playing with Fire: P) and respondent number: for example, M1A is male, he wrote for Antarctica and he was the first male composer to respond.

## Results and Discussion

We structure the results and discussion according to the three emergent themes. The first of these concerns the intended audience for the program note, including the lines of communication between composer, performer and listeners. Next, we turn to the intended role of the program note and highlight its role in relation to interpretation, understanding and collaboration. Finally, we address the content of the program note, discussing what the composers wished to communicate.

### Who the Program Note is for

Fourteen of the 17 composers felt that program notes were most often useful for both listeners and performers. Three composers (F3EW, F4EW, and F5EW) asserted that the same type of program note information could serve both performer and listener. For both groups, “the program note helps the communication between the performer and the listener” (F2A). The performer (P1) agreed that the program note could help to generate a shared understanding and that this is “crucial when the audience faces contemporary art music.” Composers who felt that program notes serve a different *function* for each cohort explained that “the languages are so different” (M2A) for performers and listeners. M4A agreed that program notes are useful for both listener and performer, but he drew a distinction between the *types* of writing for listeners and performers. This idea was expanded by three composers (M6A, M11P, and M12P) who noted the need for separate technical detail that would be relevant only for the performer and would therefore be included as performance instructions in the score.

Composer F1A felt that a program note is not relevant to the performer, advocating the need for “another kind of communication.” M7EW indicated that in his experience, the most useful communication between composer and performer is verbal dialog. M3A suggested that while information on “programmatic” (dramatic) elements of a composition might be beneficial for both performer and listener, “in the case of completely abstract music program notes can be difficult … in terms of misleading an audience” by imposing “an external framework on a work which was conceived without one.” M10P noted a similar contrast with respect to musical works that relate to extra-musical concerns such as environmental advocacy. Programmatic elements need to be shared with performers and listeners so that they can share in “larger artistic concerns that may evoke such things as landscape, literature, narrative and scientific principles … that may come to bear on the music” (M6A), thereby “allowing additional insight and a greater depth of understanding” (M7EW). Here, the program note might help the performer and listener in “navigating the piece”.

Finally, composer M8EW positioned the program note as something that might attract a performer to play the piece. He also suggested that a program note may be important in the evaluation of a work by critics or by those assessing the works for concert programming or other evaluative purposes: these cohorts often value written program notes for their insights. Associated with this were comments about the need to translate works for multiple audiences; in addition to listeners, many composers need to describe their work to external stakeholders such as funding bodies and higher education research panels. M8EW somewhat cynically suggested program notes can make a work “sound more complex/intellectual than it is [in order to] … influence their assessments favorably.”

**FIGURE 1:**
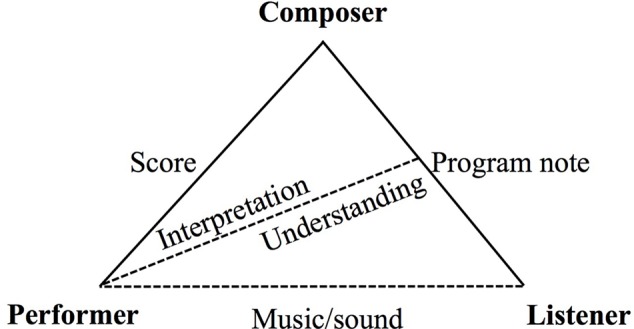
**Initial model of communication from composer through to program note and score**.

To summarize this range of views, we devised an initial diagrammatic model of communication from the composer through both program note and score. Shown as **Figure 1**, the model illustrates that while the score and the program note serve two distinct and commonly understood functions (performance instruction for the former and an aid to interpretation, understanding, and engagement for the latter), the program note carries the composer’s intentions to both the listener and the performer, developing empathy with the work. As such, it has the potential to assist performers in their interpretation (dotted line). When empathy arises, it results in co-construction of the musical experience between the composer, performer, and the listener.

In the background and facilitating this experience are the ideas of the composer or other writer, as expressed in the program note. We contend that the program note cannot be understood with a simple model of communication or transmission because the listening experience and the interpretive work of the performer are necessarily more complex than this. Indeed, these interactions offer the scope for both listener and performer to bring their own perspectives to bear on the musical experience. We explore this complexity in the next section.

### The Intended Role of the Program Note for Performers and Listeners

Participants noted several intended roles in relation to program notes. These were grouped into three themes pertaining to the program note’s influence on the listener and performer, shaping performer interpretation, and the program note as a collaborative tool.

#### The Program Note’s Influence on the Listener and Performer

In line with Scaife’s (2001) positioning of the program note as an aid to understanding, eight of the 17 composers wrote that the role of a program note is to guide listeners and performers whilst not limiting interpretation or the listening experience. This thinking was expressed in two modes: *guide* and *direction*. Each mode is expressed below using the composer’s voice.

The first mode, *guide*, highlights the composer’s desire to retain some “ambiguity or lack of meaning” (F3EW); this mode “should inspire the listener to be absorbed into listening to the music” (M9P). Reminiscent of Lorenzon’s (2015) experience noted earlier, composers wanted program notes to provide listeners with a way to access the work (F3EW) and to promote “understanding” (M8EW). The program note might include ontological information that explains, “what the piece is trying to explore or achieve” (M12P); to “inform and contextualize, but … not essentialize what a person, performer or listener ‘hears’ in the music” (M4A).

By giving “sufficient [information] to listen in a better way” (M5A), the program note in this mode offers “a subtle track … leaving some mystery in the air” and leaving the listener “ready to listen freely and without prejudices” (F1A). M3A took this further by deliberately restricting the information given to listeners so as not “to limit them in the way they might experience the work.” This view relates to the experience of composer Jean-Claude Risset, mentioned earlier, who “was excited by the variety of [listener] reactions … happy to discover occasional symbolic or even mythical evocations which I found valid and suggestive” (Cochrane, 2013, p. 29). In line with the composers whose intention was to guide the listening experience, Risset was content to suggest, rather than impose, an interpretation of his music.

Alongside this, composers wrote of the program note adding “a dimension to the work which can enable the performer and audience to become more engaged or involved in the piece” (M3A). This might communicate ontological information such as “the poetic and aesthetic suggestions of the piece” (M5A), “personal experiences of the composer … [or] other pieces of music, and/or works of art from other disciplines that informed the piece” (M7EW). Program notes might also direct “emotional/aesthetic connections and meaning and understanding of a piece for the performer” (F4EW).

The second mode, *direction*, similarly situates the program note as a “track for interpretation which has to be as generic as possible … not limiting the performer, as well as listener, on his [sic] personal interpretation of the ‘open’ meanings of musical composition” (M1A). In this mode, however, the program note is also a deliberate, two- or three-way communication tool that directs communication or generates “dialog between performer, listener, but also the composer” (M2A). This can create what the performer (P1) termed a process of empathizing with the composer’s intention.

In this mode, the program note is described as a tool that might help listeners’ and performers’ navigation of a work by communicating an ontological “sense of advocacy” (M10P). Writing of program notes as a resource, M11PS wanted to give “confidence to performers and audience alike in their exploration, interpretation and enjoyment of the music” (M11PS). Composer M11P inferred a greater level of control in order to create what he termed “ideal” performers and listeners. Similarly, within the collaborative mode M5A described an intent that was far more prescriptive, defining “all the musical details … to prevent modalities … not in accordance with the composer’s intentions.” More prescriptive composers intended the program note to direct “the way [performers] interpret the musical notes and instructions” (F5EW).

#### Shaping Performer Interpretation

Five composers intended their program notes to help shape performer interpretation and enhance performers’ understanding of their intentions. As with listeners, composers wrote of helping with a performer’s “first comprehension” of the work whilst not being “exhaustive” in relation to interpretation (M5A). For these living composers, program notes often worked alongside direct interaction “as a reminder for the performers of [contextual] elements discussed during the preparation of the pieces” (M7EW). Program notes also helped to “clarify the aesthetic approach and inspire some imaginative play” (M9P), for which purpose they included structural “information about the techniques and material of the work [to] aid in interpretation” and performance (M8EW). The performer (P1) reflected that her responsibility “is to do my best in conveying the composer’s idea. This is possible only through a close collaboration.”

Another common theme was that of “not providing too much information in the program note” (F3EW). Composers were keen to avoid a note that “explains the piece in itself” (F1A). F1A commented that his compositions were “part of my intimacy and part of my private world as creator.” F3EW’s intention was for both listener and performer to assign their own interpretations to the piece. The composer wrote of ambiguity as “one of the main attractions of music as opposed to the other art forms.” Noting the risk that listeners’ individual interpretations might be threatened, M7EW described program notes as a “double-edged sword.”

M3A acknowledged that performers and listeners might choose not to read the program notes at all. The careful balance between too little and too much information was the subject of M9P’s comments, in which he expressed the desire to “inspire the listener … often by not explaining too much, but allowing for some surprise.” Three composers asserted that, “musical works should not require a program to be effective” (M3A, M7EW, and M10P). The latter of these believed

… that the idea of reductive listening is very important … [with] many listening situations [able to] focus entirely on the sound itself, removed from any anecdotal, referential preconceptions about a piece that may be provided via program notes.

#### The Program Note as a Tool for Collaboration

For several composers, the program note represented a form of collaboration between themselves, performers and/or listeners. Mentioned above, M7EW wrote of a “recurrent and meaningful dialog with performers”. M9P emphasized co-creative freedom in this relationship: “the performer should understand what the composer is wanting to convey … but room should be left for their creative collaborative contribution.”

For F2A, listeners were initially recipients of the collaboration between composer and performer. As such, the program note “communicates some intentions from the composer that will be interpreted through the performer to finally reach the listener.” And yet this composer later extended the collaboration to include the listener: the performer “plays/acts the piece and the listener actively participates … while listening to the piece.” M9P agreed, noting listeners’ “creative contribution” to the works they hear. We term this “performative listening” in that composers, listeners and performers can be positioned as active components of the process that brings each composition to life. Their “different participation” in the process necessitates different information for each, and F2A was concerned to highlight that communication is multi-directional. Thus, the program note can be an important element in activating the listener’s participation.

The issue of collaboration was also noted in relation to the differences between information provided to the performer and the listener. F1A preferred to relay information to performers “in the process of working the piece with him or her.” M7EW agreed that some information would not be “of use or interest” to listeners. This might relate particularly to contemporary music for which performers often need explanations for new forms of notation/musical effects: “more information about techniques used in the composition” (F5EW).

Even so, F5EW’s practice was to provide a common program note for listeners and performers, as did several other composers. F3EW was adamant that performers and listeners should receive the same information, and M11P agreed: “In most cases the program note can function equally for performers and audience to illuminate their experience of the work.” Of interest, and hinting at another collaborative theme, he also noted that listeners should sometimes be kept in the dark: “if the intention of the composer is to make the performer complicit in some kind of trick on the audience, then the two will have different needs.”

### Information the Composer Communicates through a Program Note

Composers were asked what information they wanted to communicate in the program notes about their works. Their responses were analysed using the Margulis and Ferrara descriptors and are discussed below, starting with the communication with listeners.

When asked about communication with the listening audience, 12 composers responded that they include ontological (contextual) or dramatic (descriptive) information, and seven composers included both. Most composers intended this as a framework for engagement that would not “tell the audience what to hear” (M4A). Four composers suggested that semantic information was helpful and only one composer highlighted purely structural information.

Noted earlier, M3A wrote of the danger, particularly with abstract music, of creating a program note for a work conceived without an external framework. In contrast, M6A noted that program notes provide listeners of difficult or abstract music with “vital information.” Similarly, he recorded the need to communicate the “extra musical influence[s]” of programmatic music. M7EW agreed, although he felt that “ultimately the piece should work without the support of a program note.” M10P gave the specific example of electroacoustic works that essentially have no program: they “focus entirely on the sound itself, removed from any anecdotal, referential preconceptions … that may be provided via program notes.” This relates to the composer’s belief in reductive listening. However, the same composer noted the importance of communicating to performers and listeners external (ontological and/or contextual) influences.

When asked about communication with performers, two of the composers were adamant that a program note is simply not enough. In a widely varied set of responses about communicating with performers, 11 composers included structural information, 10 emphasized syntactical information, eight focused on ontological information, four introduced dramatic information, and two focused on semantic information. M11P reiterated “a range of technical information which a performer needs and which an audience other than fellow specialists does not need.” Others highlighted structural information related to “the layers of the piece” (M1A) and/or to the compositional process: “why I adopted a certain structure or what certain articulation markings have developed from” (M4A). In terms of technical notes, M10P explained that “it is important for the performer to understand the work intimately, so this does involve a more detailed/complete comprehension of the music.” M6A provided the same note to performers and listeners, but in line with MP11 he added technical information to the performance score as required. Interpretational information was rarely prescriptive, seeking rather to:

• Inspire some imaginative play or stimulus (M9P);
• Provide a track for interpretation that doesn’t limit the performer or listener (M1A);
• Help inform interpretation [with] … a more detailed analytical approach and explanation of the compositional elements (M4A);
• Help direct emotional/aesthetic connections and meaning and understanding (F4EW);
• Inform performers’ interpretation of musical notes and instructions (F5EW);
• Aid in the interpretation of the work (M8EW); and
• Give the performer a sense of what the piece aims to express (F5EW).

**FIGURE 2:**
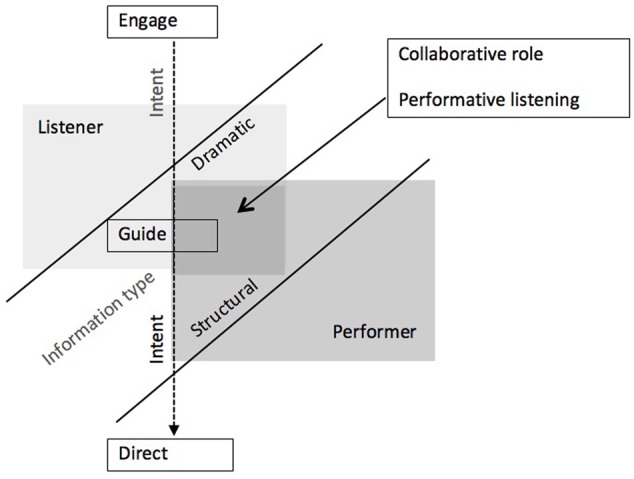
**Dimensions of composer-written program notes in which the intent may be to direct and/or engage the listener or performer by providing dramatic or structural information**.

Overall, the responses suggest that composers intend performers to receive more structural information than listeners, and that for both performers and listeners too much personal information is to be avoided. Shown at **Figure 2**, composer-written programs variously sought to engage and guide and, to an extent, to direct, both listener and performer. This was achieved through different styles of program note content engaging with collaboration through performative listening.

### Strengths and Limitations

A major strength of this exploratory study is that we formed relationships of trust with each composer, working with them from the point of commission, through the preparation process, and to the point of performance and recording. As a result, we were able to elicit frank and honest responses. Although, the study has made a contribution to understanding a very under-researched aspect of music, we must also acknowledge its limitations. First, this was a small sample of composers and we do not seek to make generalizations. We also note that this area of research is new. We uncovered many possible avenues for further research, and we hope that the model advanced in the following section will help others to continue this work.

## Conclusion

The literature confirmed that program notes are written for the concert audience, the audio or audio-visual listener, the performer, the examiner, and for Internet readers. Professional program writers, students and performers are commonly the program note writers, and they are advised to give neither too much information nor too many words. Composers, too, write program notes, and these were described as variously clear and insightful, or confounding and full of technical jargon. The literature revealed syntactical, semantic and ontological ways of listening upon repeated hearings, and it suggested that listeners may not necessarily want to receive a dramatic (descriptive) meaning that influences or even overrides their personal interpretations. Despite Margulis’s finding that dramatic notes might not increase listeners’ enjoyment, they dominate contemporary program notes and they remain an aspect recommended by educators.

Program note content typically provides a balance of historical, descriptive and structural information, and the literature exposes differing views on the inclusion of each. If the program note is situated as a form of analysis for a given work, readers can expect notes written by a work’s composer to communicate meanings that intersect between the present, and the time and world of the composer when writing the work in question.

The study reveals that composers’ intentions for their program notes vary. In this study, 14 of the 17 composers wrote their program notes for both listeners and performers. Three composers considered the same information to be relevant to both, and six felt that it should be different. Performers were often given further (mostly technical) information that was not considered relevant or interesting for the listener. Composers identified three main roles for the program note: to guide or direct the listener or performer; to shape the performer’s interpretation; and as a tool for collaboration between the composer, performer, and listener.

Eight of the 17 composers asserted that the program note should guide but not limit interpretation or the listening experience. Four of these aimed for collaboration between composer, performer, and listener intended to result in “performative listening” in which listeners were actively engaged in bringing each composition to life. Another four also encouraged a more active interpretive role for the listener resulting in the co-construction of the musical experience.

We return here to Turner’s (1933) comment that both “purely descriptive and purely technical–analytical” program notes are “objectionable and useless,” and that the overuse of dramatic writing is “useless to everybody, and positively harmful to those who are seriously trying to understand the art of music” (in Scaife, 2001, p. 7). In this study, composers favored program notes that included both dramatic (descriptive) and ontological (contextual) information for the listening audience, structural and contextual information for the performer, and specialist performance notes as required. Their comments also aligned with Bebbington’s (2004) comment that too much information is counter-productive.

**FIGURE 3:**
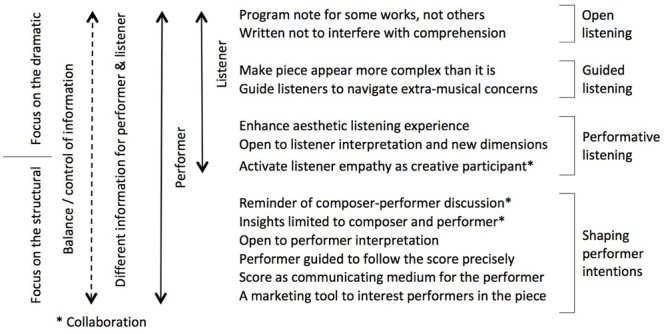
**Composer-written program notes: recipient, information type, and intended impact**.

**Figure 3** summarizes the attitudes of the composers in this study in terms of target recipient, and the role and content of the program notes. It also draws in composers’ preferences for their listeners and illustrates that the role and impact of program notes is both complex and nuanced.

The implications of this study include the need for program note writers to carefully consider the range of possibilities and intended impact of program notes, and perhaps to gauge this by seeking feedback. Similarly, the production and format of program notes for listeners merits attention. In higher education settings, commentary that recommends descriptive notes is perhaps misleading. Indeed, performers, composers and other program note writers might benefit from asking themselves when, where, how, and in what form each work is best communicated to different stakeholders. It is hoped that the range of perspectives revealed in this article will provide useful information to assist in these considerations.

### Future Research

Future research on composers’ thinking about the intent and content of program notes might focus on whether musical works are considered to require a program note, and why. This might include triangulation of data from the composer, program note content and listener. Analysis of program notes themselves, written for and by different people – composers, performers, and professional program note writers – might add further perspectives to the Goldilocks-style balance between the amount and different types of information each writer wants to communicate. Further analysis of program notes for different media or contexts might reveal whether a program note is, or should be, a different text for each reader: concert goer, CD or web listener, student performer, professional performer and so on.

Researchers might also ask under what circumstances program note information is written. If a music publisher required a note for the published version of a score, for example, would composers include information for every possible reader? If not, where might the other “versions” be kept? Similarly, if a concert promoter requested a program note, the composer would know something of the expected concert audience, whereas a CD note would have to cater for many different listening contexts. Further, do listeners want to read program notes before, during or after a performance? And finally, although this study focused on living composers, researchers might ask what happens to this knowledge—particularly that shared through collaboration—when the composer is dead? As educators and performers, we wonder whether consideration of this might prompt composers to record the information they wish to communicate in different ways. With these considerations in mind, perhaps the written program note is only one of the many communication forms we will see in the future.

## Author Contributions

The three authors confirmed that they were involved in all four authorship criteria as laid out below. Authors made substantial contributions to the conception or design of the work and the acquisition, analysis, and interpretation of data for the work; drafted the work and revised it critically for important intellectual content; approved the final version to be published; and agreed to be accountable for all aspects of the work in ensuring that questions related to the accuracy or integrity of any part of the work are appropriately investigated and resolved.

## Conflict of Interest Statement

The authors declare that the research was conducted in the absence of any commercial or financial relationships that could be construed as a potential conflict of interest.

## Footnotes

1 *Antarctica – new music for toy piano and/or piano* (2014). Pianist Antonietta Loffredo, Wirripang Pty. Ltd, Wollongong, Wirr 059. *Australia East and West: new music for viola and piano.* Violist Dawn Bennett and Pianist Diana Blom. Recording in progress. *Playing with Fire: new music for electroacoustics and live acoustic piano*. Pianist Tamara Anna Cislowska, Forthcoming with Wirripang Pty. Ltd, Wollongong.
